# Evaluation of novel recombinant antigen-based (NIE/SsIR) immunochromatographic rapid tests for *Strongyloides stercoralis*: an accuracy study

**DOI:** 10.1186/s13071-024-06569-y

**Published:** 2024-12-23

**Authors:** Salvatore Scarso, Francesca Tamarozzi, Cristina Mazzi, Monica Degani, Eleonora Rizzi, Stefano Tais, Dora Buonfrate

**Affiliations:** 1 Department of Infectious Tropical Diseases and Microbiology, IRCCS Sacro Cuore Don Calabria Hospital, Negrar Verona, Italy; 2 Clinical Research Unit, IRCCS Sacro Cuore Don Calabria Hospital, Negrar Verona, Italy

**Keywords:** Strongyloides, Strongyloidiasis, Immunochromatographic, Rapid test, Diagnosis

## Abstract

**Background:**

Strongyloidiasis is a chronic parasitic disease that results in relevant human morbidity, caused by the nematode *Strongyloides stercoralis*. This nematode has a unique and complex life-cycle. There is so far no perfect test for this helminthiasis. Rapid immunochromatographic tests (RDTs) are of interest, specifically due to their feasibility for use in the field, where public health control of strongyloidiasis is recommended. The aim of this study was to evaluate two novel RDTs, one detecting immunoglobulin (Ig) G and the other detecting IgG4, based on a combination of recombinant antigens. The primary objective was to estimate the sensitivity and specificity of these RDTs, and the secondary objective was to assess ease of interpretation.

**Methods:**

Serum samples stored in our biobank with available matched results for at least one fecal (i.e. agar plate culture or PCR) and one serology test (i.e. enzyme-linked immunosorbent assay [ELISA] or indirect immunofluorescent antibody test [IFAT]) for *S. stercoralis*, were selected for this study. Those with at least one positive result for the fecal test were considered to be true positives (irrespective of the serology), while true negatives were those with negative results for both the fecal and serology tests. The results of the RDTs were read independently by two laboratory technicians. When disagreement over the results occurred, a third reader was involved, and the final result for each test was based on consistent results from two readers. Estimates were reported along with the 95% confidence intervals (CI). Regarding the secondary objective, agreement between two independent readers was calculated with Cohen’s kappa statistic (κ).

**Results:**

A total of 90 serum samples were tested. Sensitivity of the IgG- and the IgG4-RDTs was 91.1% (95% CI 78.8–97.5) and 77.3% (95% CI 62.2–88.5), respectively. Specificity was 91.1% (95% CI 78.8–97.5) for the IgG-RDT and 100% (95% CI 92.1–100) for the IgG4-RDT. Agreement between readers was excellent (Cohen’s κ = 0.96, 95% CI 0.86–1.08%).

**Conclusions:**

The IgG-RDT demonstrated higher sensitivity and could hence be preferred for individual diagnosis, whereas the excellent specificity of the IgG4-RDT could be preferred for prevalence surveys in endemic areas. The results of both RDTs were easy to interpret based on excellent agreement between readers. Large prospective studies should follow to confirm these findings and to validate the use of either RDT for specific purposes/contexts.

**Graphical abstract:**

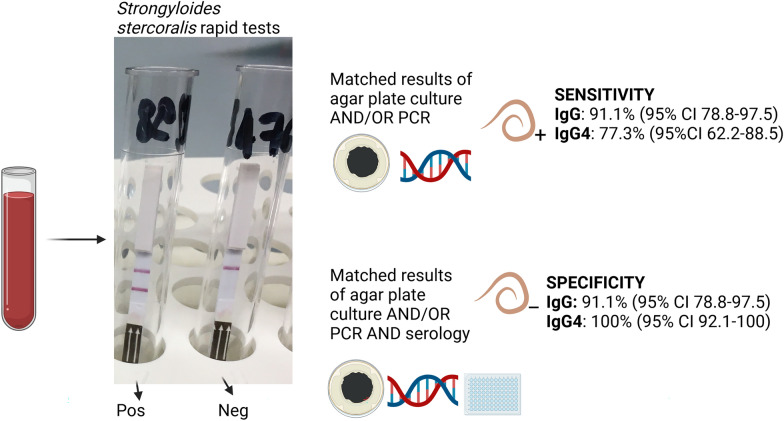

## Background

*Strongyloides stercoralis* is a soil-transmitted helminth (STH) that infects between 300 and 600 million people worldwide [1, 2]. It is mainly distributed in tropical/subtropical areas of South-East Asia, Africa, Western Pacific and Latin America. The infection is initially transmitted through the direct penetration of human skin by infective larvae found in soil contaminated with human feces, and subsequently perpetuates for decades (presumably, life-long) in the infected individuals due both to re-exposure to the infective source and to a peculiar auto-infective cycle during which larvae produced by adults in the intestine re-infect the same individual [3]. Strongyloidiasis often goes unnoticed due to intermittent, non-specific symptoms; eosinophilia is often but not always present. In cases of immunosuppression, larval load can increase dramatically, and all parasite stages can be found throughout the body. This condition, known as hyperinfection or dissemination, is most often fatal [4].

Despite the potential harm inflicted by this nematode, the clinical burden and the prevalence of this parasitic infection have long been underestimated, mostly due to diagnostic issues [5]. Classical copromicroscopy methods for other intestinal parasites (e.g., Kato-Katz, formol-ether concentration) show an extremely low sensitivity for *S. stercoralis*. Other stool-based tests that are more specific for the detection of larvae, such as agar plate culture (APC) and the Baermann method, as well as PCR, while more sensitive than stool microscopy, still have unsatisfactory sensitivity [6]. Moreover, PCR is at present too expensive for large-scale use in endemic areas, and the Baermann method and APC are time-consuming and require good microscopy skills [7]. Serological methods have demonstrated the best sensitivity to date, but the results differ substantially between assays. Commercially available serology assays are mostly enzyme-linked immunosorbent assays (ELISAs) based on either crude or recombinant antigens [6]. The use of recombinant antigens aims to improve test reproducibility, scalability and specificity, although the latter characteristics for recombinant antigen-based assays for *S. stercoralis* evaluated so far are variable [6].

In addition to seroassays using the ELISA format, a number of immunochromatographic tests (ICTs) have also been developed recently, most of which are based on the recombinant antigens NIE [8] and SsIR [9–15]. The rapidity at which these tests can be carried out and results obtained, as well as the ease-of-use with no need of microscope skills or laboratory equipment, make ICTs of particular interest in different settings, including low-middle income countries. Rapid diagnostic tests (RDTs) would also be attractive options in non-endemic countries, in particular for pre-transplant screening (possibly in association with fecal-based tests) [16, 17].

Two immunochromatographic RDTs, based on a combination of two recombinant antigens (NIE and SsRI) and targeting either immunoglobulin (Ig) G or IgG4 antibodies, have recently been developed by InBios International Inc. (Seattle, WA, USA). The combined use of two recombinant antigens might increase the performance of a test, compared to similar diagnostics based on single antigen. As such, it would be relevant to compare these IgG and IgG4 assays to evaluate the best format in clinical practice.

In this study we perform a preliminary evaluation of the diagnostic performance of these two novel NIE/SsIR-based RDTs for the diagnosis of *S. stercoralis* infection.

## Methods

### Study design and objectives

This study was a single-center, retrospective study that used sera available in the biobank (“Tropica Biobank”) of the IRCCS–Sacro Cuore Don Calabria hospital, a referral center for parasitic diseases in Italy. The primary objective was to assess the sensitivity and specificity of the NIE/SsIR RDTs for the diagnosis of *S. stercoralis* infection in migrants from endemic countries who attended the Department of Infectious, Tropical diseases and Microbiology (DITM) of the IRCCS–Sacro Cuore Don Calabria hospital, Negrar, Verona, Italy. The secondary objective was to evaluate the ease of interpretation of the results of the novel RDTs. As exploratory analyses, we evaluated: (i) the performance the RDTs when performed on samples of whole blood and (ii) the stability of the test result 1 h after being performed.

### Sample selection

Serum and blood samples were selected from the database of the Tropica Biobank; all samples in this biobank are stored at - 80 °C and originate from patients who provided informed consent in writing to donate their samples for research purposes. For the serum and/or blood samples to be included in the study, the following eligibility criteria held: (i) sample was collected from a migrant originating from a country endemic for *S. stercoralis* (i.e. countries of Africa, Latin America, South-East Asia and western Pacific regions) and (ii) results were available for both a fecal test and at least one serology assay for *S. stercoralis* performed ± 30 days from the collection of the serum (routine tests performed at DITM are described in section Routine analyses for strongyloidiasis and sample classification). Exclusion criteria were: (i) insufficient serum volume and (ii) treatment with ivermectin in the previous 6 months.

### Test procedures

The Two RDTs were available as dipsticks, which consist of a test strip (membrane) that is pre-coated with *S. stercoralis* antigens on the test line region. When a line appears on the test region, the result is positive. A separate control line is present on the membrane, and this line should always be present to consider the test valid. The two RDTs were tested in parallel on the same samples, although one sample was tested with the IgG version only. Before performing the test, sera were re-coded by persons not directly involved in the laboratory procedures. The RDTs were performed in accordance with the manufacturer’s instructions (InBios International Inc.). Briefly, samples were first thawed at room temperature; then 15 µl of a sample was added to the sample pad of a dipstick, and the dipstick placed upright into a tube containing 3 drops of the buffer included in the test kits. The results were read after 15–20 min, as per the manufacturer’s instructions, and at 1 h after the first reading, in the context of this exploratory study. The laboratory staff who performed and read the tests were blinded to the results of any test performed previously. Two independent readers reported the test results independent of each other. The result was considered to be invalid in the absence of a control line. The presence of both control and positive lines was defined as a positive test result; the presence of the control line only was defined as a negative test result. Equivocal results (positive control line, but readers were uncertain about the test line) were classified as negative for the purpose of the study.

A third reader was consulted when the two readers disagreed on the test results. Further, a fourth reader, blinded to the interpretation of the other readers, read the results 1 h after the first reading.

### Routine analyses for strongyloidiasis and sample classification

The results of the RDTs were compared to the results of the tests for strongyloidiasis that had previously been routinely performed at the study site. These latter test include fecal tests (APC and PCR) and serology assays (an in-house immunofluorescence test [IFAT] and a commercial ELISA). When *S. stercoralis* larvae were observed on copromicroscopy after formol-ether concentration, the result was also recorded for sample classification. The procedure for APC follows a modified Koga agar plate method, and previously showed 45.4% (95% confidence interval [CI] 30.4–61.1) sensitivity [18]; the specificity of microscopy methods can be considered to be 100% since they are performed by expert microscopists. The PCR is a real-time assay based on Verweji’s method and has been described previously [19]. It has been evaluated in several studies in different settings, with average reported sensitivity and specificity of around 70% and 87%, respectively [6]. The IFAT is an in-house method, with 94.6% (95% CI 90.7–98.5%) sensitivity and 87.4% (83.4–91.3%) specificity [20]. The commercial ELISA used is *Strongyloides ratti* IgG ELISA (Bordier Affinity Products SA, Crissier, Switzerland), which detects IgG antibodies against somatic antigens from larvae of *S. ratti*. A recent prospective study estimated its sensitivity and specificity at 65.9% (95% CI 53.4–69.0%) and 100% (95% CI 99.8–100%), respectively[7].

True positive cases were defined as samples with at least one positive fecal test based on APC, formol-ether microscopy and PCR. True negative cases were defined as samples with negative results for fecal test(s) and serology.

### Statistical analysis

#### Sample size

For sample size calculation, we referred to previously published data on the other NIE/SsRI assays produced by the same company in ELISA format [21], and assumed that the new RDTs would not perform any worse. In the case of the worst performing assay previously evaluated (detecting IgG4), for a sensitivity of 70% and specificity of 97%, the width of the 95% CIs were 26% (55–82%) and 13% (86–99%), respectively, when including 50 true positive and 50 true negative samples.

#### Data analysis

Test results were presented in contingency tables from which parameters such as sensitivity (positive RDT results for true positive samples), specificity (negative RDT results for true negative samples) were calculated. Estimates were reported along with the 95% CIs. The McNemar test was used to compare the sensitivity and specificity of the two RDTs.

For the secondary objective, the agreement between the two independent readers was calculated using Cohen’s kappa statistic (κ coefficient). The values of the coefficient were interpreted as follows: ≤ 0 as no agreement; 0.01–0.20 as none to slight agreement; 0.21–0.40 as fair agreement; 0.41–0.60 as moderate agreement; 0.61–0.80 as substantial agreement; and 0.81–1.00 as almost perfect agreement.

Exploratory analyses were analyzed as follows: (i) a descriptive report of the number of either of the RDTs with positive results when tested with whole blood and (ii) the results of either RDT read 1 h after the initial reading was compared with the results at the recommended reading time using the McNemar test.

## Results

**Fig. 1:**
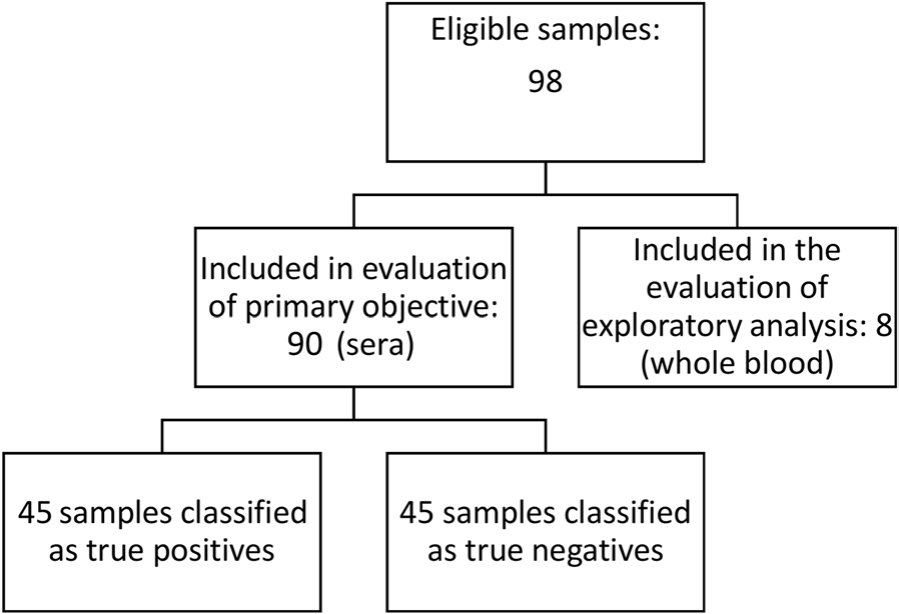
Study flow chart

A total of 98 samples (90 serum and 8 whole blood specimens) were tested (Fig. 1). Of the serum samples, 45 were classified as true positives, and the other 45 as true negatives. Of the 98 samples, 97 were tested with the IgG4-RDT and 98 were tested with the IgG-RDT. For the primary objective, we analyzed only the results of the serum samples. The results of the RDTs in comparison with the panel of fecal tests and the sensitivity and specificity of the novel assays are reported in Table 1.

**Table 1:** Results of the rapid diagnostic tests, and sensitivity and specificity calculated against the panel of fecal tests

Rapid diagnostic test	RDT result	Positive in fecal tests	Negative in fecal tests	Sensitivity of RDT (95% CI)	Specificity of RDT (95% CI)
IgG-RDT	Positive	41	4	91.1% (78.8–97.5%)	91.1% (78.8–97.5%)
	Negative	4	41		
IgG4-RDT	Positive	34	0	77.3% (62.2–88.5%)	100% (92.1–100%)
	Negative	10	45		
*P*-value^a^				0.014	0.045

*Ig *Immunoglobulin,* CI* confidence interval,* RDT* rapid diagnostic test

^a^McNemar test; IgG versus IgG4 for sensitivity and specificity

There were two and zero equivocal results, which were classified as negative, for the IgG-RDT and the IgG4-RDT, respectively.

Agreement between readers was excellent Cohen’s κ = 0.96 (95% CI 0.86–1.08%).

At the second reading, at 1 h after the initial reading, one originally positive IgG-RDT strip turned negative, and one originally negative IgG4-RDT turned positive. No statistically significant differences were found between results read at the recommended timeframe and 1 h later.

The subgroup of RDTs run on blood were all tested against true positives only. There were one negative and seven positive results for the IgG-RDT, and six positive and two negative results for the IgG4-RDT. There was one equivocal result for the IgG-RDT and two equivocal results for the IgG4-RDT.

## Discussion

**Fig. 2:**
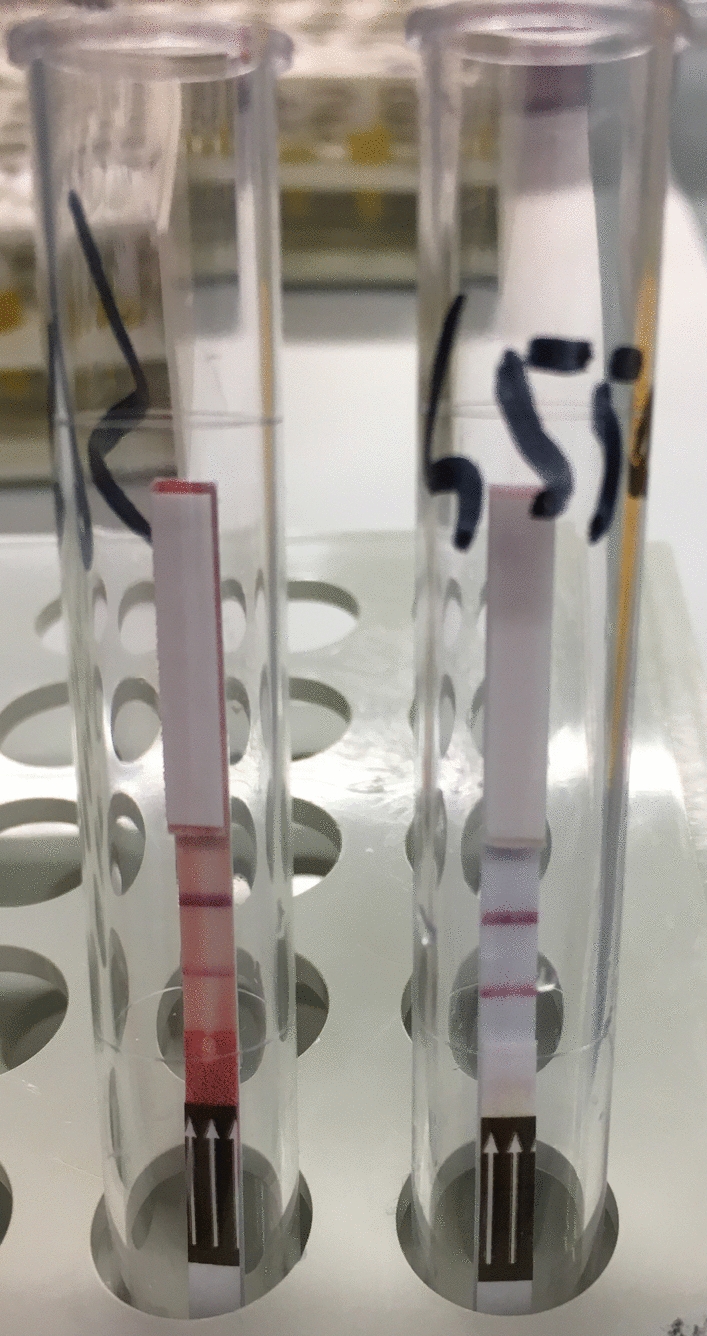
Positive whole blood (dipstick on the left) and serum (dipstick on the right) samples, as demonstrated by the presence of both control and test lines

In this study, the RDT based on IgG demonstrated significantly higher sensitivity but lower specificity than the IgG4-based RDT. The interpretation of these RDT results was generally straightforward, as demonstrated by the excellent agreement between readers and few equivocal results, mainly observed when using whole blood due to a darker background (Fig. 2).

The results obtained in this study are in line with those obtained with the ELISAs from the same manufacturer that are based on the same antigens, similarly showing higher sensitivity when detecting IgG (IgG ELISA: 92%, 95% CI 88–97%) than when detecting IgG4 (IgG4 ELISA: 81%, 95% CI 74–87%) and higher specificity when detecting IgG4 (94%, 95% CI 91–97%) than when detecting IgG (91%, 88–95%) [21]. The use of either RDT (detecting IgG or IgG4) might be suitable for different cases. A highly sensitive test is generally more suitable for individual screening purposes, such as in the transplant setting, where the goal is to avoid missing any infection [6]. In such a setting, both donors and recipients might be screened using the most sensitive test or a combination of tests to mitigate the risk of disease dissemination in transplant candidates [17]. Treatment of patients with possible false positive results would be acceptable, given the excellent tolerability profile of ivermectin [22] against the harm (potentially fatal) of not treating a missed infection. Conversely, for prevalence estimates in the context of control programs in endemic areas, high specificity is preferred [23]. In this context, treatment is administrated to all of the target population if prevalence is estimated to be above a pre-defined threshold; as the performance of the assay used has already been taken into account in this setting, precise individual diagnoses are not required [23]. The WHO guidelines recommend methods such as the Baermann method or APC for the evaluation of prevalence at the population level and for public health control of strongyloidiasis, but acknowledge the possible use of antibody testing if an assay proves sufficiently accurate [24]. In this context, the IgG4-RDT assessed in the present study would be more suitable than the IgG-RDT. However, further evidence from larger cohorts and studies in a field setting are necessary to confirm these results and validate them for broader use. Such future studies should also include the proportion of equivocal results in order to understand whether such test would be acceptable especially for finger-prick blood deployment.

Another interesting feature of the RDTs assessed here was that in our study the results remained stable long (1 h) after the recommended reading time. While it is always preferable to adhere to the given timeframe for reading, if confirmed on a larger cohort, the stability of the results for some time could be appealing when the assays are used in large-scale screenings, where it may be challenging to read the results at the exact time stipulated by the manufacturer.

The development of these RDTs in a cassette format to enhance ease-of-use in the field could also be considered, although the higher costs of the device in this format and potential concerns regarding plastic disposal should be considered. All of these aspects should be taken into account, together with potential use in control programs integrating more than one disease, when performing a cost-efficacy analysis comparing fecal assays, point-of-care assays and assays to be performed in a centralized lab.

Other ICTs developed for the diagnosis of strongyloidiasis have shown variable performance, mostly in retrospective, laboratory-based studies. An RDT prototype based on the NIE/SsIR combination [9] was initially evaluated in laboratory-based studies and subsequently as a NIE-based IgG4 test in a cassette format; the latter demonstrated variable performance in both retrospective, laboratory-based studies (sensitivity 82.4–97%, specificity 73.8–94.5%) [11, 12] and prospective studies (sensitivity 79.4–93.9% and specificity 93.6%) [7, 14]. An immunochromatographic IgG test based on a crude *S. stercoralis* somatic antigen was evaluated in a single laboratory-based study, showing a sensitivity and specificity of 93.3% and 83.7%, respectively [13]. The same group of authors then developed two SsIR-based immunochromatographic tests, one detecting IgG and one targeting IgG4. In a laboratory-based study, these authors found the IgG test to have a higher sensitivity than the IgG4 test (91.7% vs 78.3%) with specificity of 84.8% versus 83.8% [10]. Clearly, comparative studies that use in parallel the available RDTs (and possibly ELISAs) are needed for robust validation of assays’ performance and for defining their role in screening and/or individual diagnosis.

Among the limitations of this study are the lack of data on the immunological status of the participants and co-infections. A prospective study is required to further evaluate these promising RDTs.

## Conclusions

Despite the limitations derived from the study design, this preliminary evaluation of the novel NIE/SsIR RDTs showed promising results. The RDT that detected IgG may be more appropriate for individual screening, whereas the RDT detecting IgG4 might be suitable for deployment in public health control activities. Both RDTs were easy to use and the results were easy to interpret, although whole blood use should be evaluated in larger cohorts. Overall, further studies are required to confirm these findings and validate these RDTs in the appropriate contexts.

## Data Availability

The raw data are available in Zenodo, at: https://doi.org/10.5281/zenodo.14017717.

## Abbreviations

APC: Agar plate culture
DITM: Department of Infectious Tropical Diseases and Microbiology
ICT: Immunochromatographic test
RDT: Rapid diagnostic test
STH: Soil-transmitted helminth
